# Spatial heterogeneity affects predictions from early-curve fitting of pandemic outbreaks: a case study using population data from Denmark

**DOI:** 10.1098/rsos.220018

**Published:** 2022-09-14

**Authors:** Mathias L. Heltberg, Christian Michelsen, Emil S. Martiny, Lasse Engbo Christensen, Mogens H. Jensen, Tariq Halasa, Troels C. Petersen

**Affiliations:** ^1^ Niels Bohr Institute, University of Copenhagen, Blegdamsvej 17, Copenhagen E 2100, Denmark; ^2^ Laboratoire de Physique, Ecole Normale Superieure, Rue Lhomond 15, Paris 07505, France; ^3^ Infektionsberedskab, Statens Serum Institute, Artillerivej, Copenhagen S 2300, Denmark; ^4^ DTU Compute, Section for Dynamical Systems, Department of Applied Mathematics and Computer Science, Technical University of Denmark, Anker Engelunds Vej 101A, Kongens Lyngby 2800, Denmark; ^5^ Animal Welfare and Disease Control, University of Copenhagen, Gronnegårdsvej 8, Frederiksberg C 1870, Denmark

**Keywords:** pandemics, agent-based modelling, spatial heterogenity, fitting, COVID-19

## Abstract

The modelling of pandemics has become a critical aspect in modern society. Even though artificial intelligence can help the forecast, the implementation of ordinary differential equations which estimate the time development in the number of susceptible, (exposed), infected and recovered (SIR/SEIR) individuals is still important in order to understand the stage of the pandemic. These models are based on simplified assumptions which constitute approximations, but to what extent this are erroneous is not understood since many factors can affect the development. In this paper, we introduce an agent-based model including spatial clustering and heterogeneities in connectivity and infection strength. Based on Danish population data, we estimate how this impacts the early prediction of a pandemic and compare this to the long-term development. Our results show that early phase SEIR model predictions overestimate the peak number of infected and the equilibrium level by at least a factor of two. These results are robust to variations of parameters influencing connection distances and independent of the distribution of infection rates.

## 1. Introduction

Over the past years, the pathogen now known as SARS-CoV-2 has spread dramatically, risen in several waves, paralyzing societies, resulting in a large number of deaths and severe economic damage worldwide [1,2]. Mathematical models have estimated the reproduction number and guided the authorities in an attempt to minimize the damage caused by the virus [3–6]. Even though modern algorithms using machine learning have helped the process [7,8], the majority of models used to predict the size of the pandemic (or a rising wave of the disease) have been variants of the SIR/SEIR model. The SIR model was originally proposed in 1927, in the seminal work of Kermack and McKendrick, who successfully described the evolution of a pandemic, using a mean field approximation where all individuals are described as one population [9]. In the investigations of the SARS-CoV-2 pandemic, the mathematical models have varied in complexity including simple deterministic compartmental models [6,10], meta-population compartmental models [11–13], individual based models without including spatial specifications [4,14,15] and spatio-temporal agent-based models [16].

One aspect in the modelling is the ability to predict the infection peak height and the number of individuals who will be infected based on the early rise in the number of infected (before governmental interference). Earlier work has pointed out the importance of including heterogeneity when modelling the spread of infectious disease such as contact patterns between individuals [17], population mixing assumptions [18], heterogeneities caused by super-spreaders [15], and the spatial dependency of COVID-19 [19,20]. These mathematical models have not combined heterogeneous elements nor quantified how much the early SIR/SEIR predictions might be biased.

In this paper, we include geographical distributions based on an entire population, using population data of Denmark. When the SIR model was originally formulated, 95 years ago, data was not available to investigate the effects of geographical and demographic differences among the population, which might be one of the reasons why fundamental properties for diseases, such as the basic reproduction number (*R*_0_), can vary significantly between different regions [21]. However, with modern collection of data, these geographical aspects might be accounted for. Our main goal of this work is therefore to investigate the importance of heterogeneities in a geographically distributed population on the spread of a pandemic. We find that the heterogeneity arising from spatial inhomogeneities causes an increase in the early stage of the pandemic, affecting the initial forecast and highlighting the importance of early intervention in order to minimize the effects of the pandemic.

### 1.1. Construction of the model

**Figure 1.**
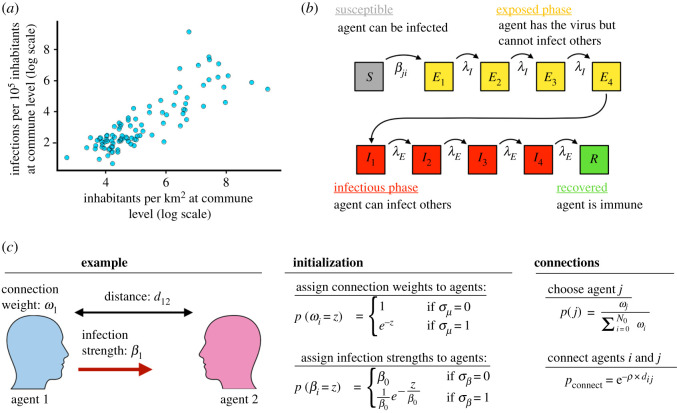
(*a*) Population density (*x*-axis) and the number of infections per 10^5^ inhabitants (*y*-axis) for each commune in Denmark. (*b*) Illustration of the modified susceptible-exposed-infected-removed (SEIR) model used. It consists of 10 consecutive states (*S*, *E*_1−4_, *I*_1−4_ and *R*), with transition rates governed by *β*, *λ*_*E*_ and *λ*_*I*_, respectively. (*c*) Illustration of how the spatial network is generated and heterogeneities in individuals included.

In order to investigate the effect of a geographically distributed population, we extracted the number of infected per commune (from the Danish Serum Institute [22]) and divided this number with the number of inhabitants in each commune to obtain the number of infected per individual in each commune. This number we then plotted against the number of inhabitants in that specific commune (extracted from statistics Denmark [23]). Doing so, we found a strong correlation between the population density and the number of infections per inhabitant as seen in figure 1*a*. This observation has been made for many other countries [24–29] and underlines the aspect of disease spreading that has been observed since ancient times; that densely populated regions often have larger pandemics than the rural areas. Note that in the very early stage of a pandemic, before the exponential growth rate is reached, micro outbreaks will guide its evolution and these events can likely take place in regions with low density [30].

### 1.2. Disease simulation

To simulate evolution of the disease, we assigned each individual (agent) to a state (predominantly initialized in state *S*) and assigned four states to the exposed phase and four states to the infectious phase, in order to achieve an Erlang distribution (which is related to the Gamma distribution) of time in each phase [31]. Once in the exposed phase, the infected agent has a rate to move into another state, where the rate is fixed based on experimental data in order to achieve a mean time in the exposed phase of approximately 4 days (table 1). Each agent in the Infectious phase can infect other agents that have a connection to this agent in the network. This definition of agents in discrete states is naturally a simplification of the real pandemic, and we stress that this mathematical model aims at describing the spread of the disease in a simple way that does not capture all aspects of the real disease. We do not believe that this impacts our main conclusions in any way, as we are aware that one should always be careful when making these kinds of simplifications. To investigate the effect of infection heterogeneities, we assigned an infection strength to each connection in the network, so some agents were more infectious than others. In order to control the degree of this heteogeneity, we assigned a boolean parameter $\sigma_{\beta }$, that if switched on generated an exponential distribution in infection strengths, keeping the mean field reproduction number fixed. The reproduction number between the ABM and the SIR model is related through the parameter $\tilde{\beta }=\beta (\mu /2N_{0})$. All transitions between states and infection of other individuals were done using the Gillespie algorithm [32]. This is schematized in figure 1*b*.

**Table 1.** Overview of the 10 parameters applied in this study, their typical value, and the ranges we have considered. The first six parameters are standard SEIR parameters, whereas the last four parameters define the heterogeneity in the model. These four parameters do not affect the SEIR model.

variable	description	value	range	units
*N*_0_ :	population size	5.8 × 10^6^	10^5^−10^7^	—
*N*_init_ :	number of individuals initially infected	100	1−10^4^	—
*μ* :	average number of network contacts	40	10−100	—
*β* :	typical infection strength	0.01	0.001−0.1	d^−1^
*λ*_*E*_ :	rate to move through $\frac{1}{4}$ of latency period	1	0.5−4	d^−1^
*λ*_*I*_ :	rate to move through $\frac{1}{4}$ of infectious period	1	0.5−4	d^−1^
$\sigma_{\mu }\;:\;$	population clustering spread	0	0−1	—
$\sigma_{\beta }\;:\;$	interaction strength spread	0	0−1	—
*ρ* :	typical acceptance distance	0.1	0−0.5	km^−1^
$\epsilon_{\rho }\;:\;$	fraction of distance-independent contacts	0.04	0−1	—

### 1.3. Network creation

In order to construct the underlying network, we created a set-up whereby two agents were chosen at random but based on their individual connectivity weight each iteration and connected with some probability based on their spatial position. To include the possibility of highly connected individuals independent of their spatial position, we assigned a boolean parameter $\sigma_{\mu }$ that, if switched on, generated an exponential distribution in weights for the individuals, keeping the mean field reproduction number fixed similar to the heterogeneity in infection strengths. To include the spatial position in the network, we introduced a parameter *ρ*, so the probability of connecting two chosen agents decayed exponentially with the distance between them: $p_{\mathrm{connect}}=e^{-\rho \times d_{ij}}$. In order to allow some long-distance connections we introduced another parameter *ε* ∈ [0; 1], that determines the fraction of distance-independent contacts. To construct the network of spatially distributed contacts, we chose the parameters using data based on:

— The geographical location of people in Denmark (from Boligsiden [33])
— The average number of contacts per individual per day of 11 (from HOPE [34]). Given an average infectious period of 4 days, we approximate the average number of effective contacts to be *μ* = 40
— The average commuting distance *ρ* = 0.1 km^−1^ and the fraction of long-distance commutes $\epsilon_{\rho }=4\text{\%}$ (from statistics Denmark [23])

This is schematized in figure 1*c* and further described in the Methods section. All 10 parameters in this model are defined and outlined in table 1. We note that in order to keep the parameters space low, this model does not include the effects of temporal changes such as seasonality and holidays. While all agents have been assigned parameters to their infection network that are derived from statistics of Denmark for both employees and students, we have not divided each agent into specific occupations.

Before including heterogeneity, we compared the ABM to the corresponding SEIR model as a test, and found them to agree within 5% for all parameter configurations tested. Here, we also tested the effect of the number of individuals initially infected (see electronic supplementary material). This concludes that the SEIR and ABM model are calibrated to have the same reproduction number in the absence of heterogeneities. Next, we will introduce heterogeneities into the system, while keeping the sum of contacts and infection strengths constant, to study how this affects the evolution of the pandemic.

## 2. Results

### 2.1. Geographical distributions in a population and large variances in numbers of contacts leads to increased infection levels

**Figure 2.**
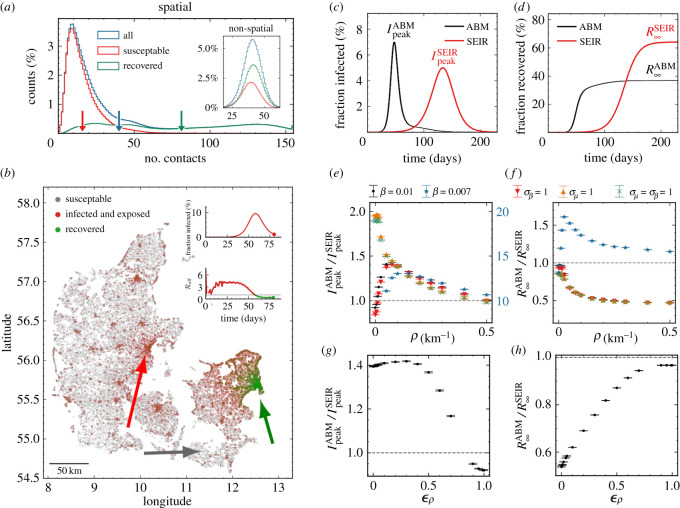
(*a*) Histograms showing the number of susceptible (red) and recovered (green) individuals at the end of an epidemic with *ρ* = 0.1 km^−1^. The distribution before the epidemic is shown in blue. The arrows show the mean of each distribution. The inset shows the same for *ρ* = 0 km^−1^. (*b*) Visualization of the spatial position of individuals during the infection and which state they are in. Green arrow: largest city in Denmark (Copenhagen): mostly recovered. Red arrow: Second largest city in Denmark (Aarhus): mostly infected. Grey arrow: low-population area: mostly susceptible (i.e. have not been infected). (*c*) Number of infected individuals as a function of time. Data shown for the spatially distributed network (*ρ* = 0.1 km^−1^). Simulation was repeated 10 times. (*d*) Cumulative sum of individuals who have had the disease as a function of time (with *ρ* = 0.1 km^−1^). (*e*) Relative difference in maximal number of infected, *I*_peak_, between deterministic (SEIR) and ABM as a function of *ρ*, and shown for different parameters. Note the data for *β* = 0.007 are shown in blue with a factor 10 scaling (right *y*-axis). (*f*) Relative difference in total number of infected at the end of the epidemic, *R*_∞_, between deterministic (SEIR) and ABM as a function of *ρ*. Colours similar to (*e*). (*g*) Same as (*e*), but as a function of $\epsilon_{\rho }$. (*h*) Same as (*f*), but as a function of $\epsilon_{\rho }$.

Having introduced heterogeneity, the distributions of connections in this network were created automatically through the population clustering, see figure 2*a*. This naturally leads to individuals living in densely populated areas having higher number of connections. In an example simulation with 100 initially infected individuals, *N*_init_ = 100, we observed a spatial difference in areas affected by the disease (figure 2*b*), as expected. Note that we also show the effective reproduction number ($R_{\mathrm{eff}}$) as a function of time in the lower part of the inserted panel. One region reached local endemic steady state (green arrow, figure 2*b*) while other regions of similar density were highly infected (red arrow, figure 2*b*) and yet other districts were almost unaffected (grey arrow, figure 2*b*). To quantify the effect of population clustering, we compared the ABM result to the reference SEIR model of similar parameters. Generally, we observed that the epidemic developed faster with a higher infection peak *I*_peak_, but also subsided quicker, leading to a lower number of infected once reaching endemic steady state, *R*_∞_ (figure 2*c*,*d*).

In order to explore how population clustering affects the epidemic, we chose a reference value of infection rates, *β* = 0.01, and an alternative value of *β* = 0.007. In the absence of spatial dependence (*ρ* = 0 km^−1^), these correspond to initial reproduction numbers $R_{0}\approx 1.7$ and 1.1, respectively. Here, we define the reproduction number as the average number of agents each infectious agent will infect in the first part of the disease. Increasing the spatial dependence (i.e. increasing *ρ*) leads to a significant rise in the infection peak for the ABM, $I_{\mathrm{peak}}^{\mathrm{ABM}}$, compared to the (unaffected) SEIR model, $I_{\mathrm{peak}}^{\mathrm{SEIR}}$ for both the reference value and the alternative lower value of *β* (black and blue points, figure 2*e*). We introduced heterogeneity in infection strengths ($\sigma_{\beta }=1$, see figure 1*b*), thus making some individuals much more infectious than others (i.e. including *super shedders*). We found no significant impact from this effect (red points in figure 2*e*). Similarly, we introduced heterogeneity in connection weights ($\sigma_{\mu }=1$, see figure 1*b*), thus making some individuals much more likely to form contacts than others (i.e. including *super connecters*). This leads to a significant effect for *ρ* = 0 km^−1^, which converges towards the other curves for *ρ* > 0.1 km^−1^ (orange (only super connecters) and green (super connecters and super shedders) points in figure 2*e*). The total number of individuals that have been in the infectious state, when there are not enough susceptible agents for the disease to keep infecting new individuals, is termed *R*_∞_, and this converged towards half of the SEIR model prediction as a function of *ρ* except for *β* = 0.007 where the endemic steady state level is larger than the one obtained by the SEIR model (figure 2*f*). We note that in reality, individuals can lose immunity and therefore new waves can emerge. But for a completely susceptible population, *R*_∞_ describes the fraction of the population that will get the disease during a specific wave. Fixing *ρ* = 0.1 km^−1^ and increasing the fraction of distance-independent contacts, $\epsilon_{\rho }$, we found that $I_{\mathrm{peak}}^{\mathrm{ABM}}$ is almost unaffected for $\epsilon_{\rho }<0.5$ (figure 2*g*), while $R_{\infty }^{\mathrm{ABM}}$ increases linearly towards the SIER model $R_{\infty }^{\mathrm{SEIR}}$, as expected (figure 2*h*).

### 2.2. Fitting early infection curves leads to significant bias in estimating the size of the pandemic

**Figure 3.**
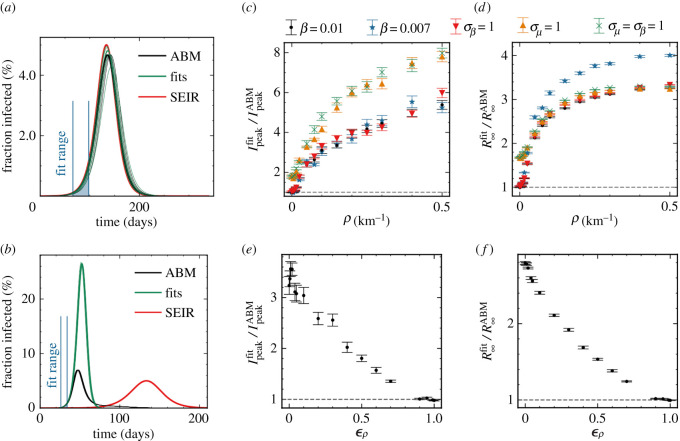
(*a*) Number of infected individuals for the ABM in black, the SEIR model in red and the SEIR fits to the ABM data in green. Blue lines show the interval where parameters are fitted (also shown below the curves). Here, *ρ* = 0 km^−1^. (*b*) Same as (*a*) but with population clustering (*ρ* = 0.1 km^−1^). (*c*) Relative difference in maximal number of infected, *I*_peak_, between the fit and the ABM for different values of *ρ*. Simulations repeated 10 times for each data-point. (*d*) Relative difference in total number of infected at the end of the epidemic, *R*_∞_, between the fit and the ABM for different values of *ρ*. (*e*) Same as (*c*), but as a function of $\epsilon_{\rho }$. (*f*) Same as (*d*), but as a function of $\epsilon_{\rho }$.

Next, we consider how these heterogeneities bias the traditional SEIR model predictions, especially the predictions based on fits to the number of infected (i.e. the curve to be flattened) in the beginning of the epidemic (see Methods). Without spatial dependence, the predicted curves fitted the number of infected individuals very well (figure 3*a*). Introducing spatial dependence (*ρ* = 0.1 km^−1^) leads to a severe overestimation of the epidemic based on the number of early infection cases (figure 3*b*). This result can be interpreted by the fact that in societies where population density and thus individual contact number varies significantly, the early phase will be driven by people with many contacts (*super connecters*). This typically happens in cities where the population density is high. Increasing the spatial dependence *ρ*, we found that the SEIR model predictions overestimated the infection peak height *I*_peak_ and the total number of infected *R*_∞_ significantly even for very small spatial heterogeneities (figure 3*c*,*d*). We observed this general trend for all tested combinations of parameters and heterogeneities. In particular, we found that if long-distance connections $\epsilon_{\rho }$ are below 10%, the bias in the estimated infection peak height, *I*_peak_, was constant within statistical uncertainty (figure 3*e*). For the total number of infected, *R*_∞_, we observed an almost linear regression to the SEIR model as $\epsilon_{\rho }$ approaches one. However, even when $\epsilon_{\rho }=0.25$, the prediction bias was still a factor of two (figure 3*f*). We concluded from these curves a general trend; if one fits an SEIR model to infection numbers during the beginning of an epidemic, and use these estimates to predict the characteristics of the epidemic at a national level, one overestimates the number of infected by at least a factor of two.

## 3. Discussion

In summary, this work outlines that the degree of population clustering in Denmark creates a discrepancy between the early predictions made by the SEIR models and the underlying agent-based interactions. It results in a significant overestimation of the impact of the disease, both in terms of maximal number of simultaneously infected (by a factor of 3) and the endemic steady state level (by a factor of 2.5). Such discrepancies have been observed for earlier pandemics, for instance, the 1918 Spanish flu, where the predicted number of individuals that would get the disease within a season turned out to be higher than the actual outcome [35]. The present results can be an important element in explaining these mismatches, even though other elements, such as for instance social distancing and the population behaviour, play a vital part. When facing a rising pandemic, societies are faced with the task of laying out strategies to minimize the consequences, including the importance of *flattening the curve*. While this is truly crucial to avoid overpopulated hospitals, the understanding of the pandemic should be taken seriously enough that we might specify to a higher degree of certainty which curve to be flattened. Our results highlight an important element in the prediction of infection levels and quantify the effect of density heteogeneities. We are aware that these results are not directly applicable to the pandemic of SARS-CoV-2 as a whole, since numerous mutations have increased the infection rates compared to the early estimates and created a strong heterogeneity in the infection worldwide. Furthermore, the actual evolution of the pandemic was highly affected by the different governmental interventions, that are not included in this work. However, this study emphasizes the abnormally large reproduction rates in the beginning of a pandemic, due to individuals with more connections than the rest of the population and attempts to quantify this bias, when countries should estimate the severity of a disease based on the data collected in the early phase. This also underlines the benefits by making lockdowns early in the pandemic, when a population is highly susceptible (for instance to a new mutation) and therefore can be driven by *super connectors*. Since people living in city-clusters are more likely to have many contacts, or infection events, they are on average more likely to be affected in the early stage of the pandemic (if they do not implement social distancing). By removing contacts from these individuals, through some level of interaction in order to reduce the number of social contacts, one can avoid the worst peak while affecting the lowest number of people. While our work describes some fundamental aspects of the disease spreading, this model does not consider asymptomatic individuals, which has been an important aspect of the SARS-CoV-2 pandemic [36,37]. Effectively, asymptotic individuals would correspond to a very heterogenous distribution of time the agents spend in the infectious state. While agents with symptoms would predominantly isolate themselves and thereby significantly reduce their ability to infect other agents, asymptomatic agents would have a long time in the infectious state, thereby infecting more individuals. In this work, we have not considered the observation that individuals lose their immunity to SARS-CoV-2 which was first studied in the Brazilian city of Manaus. For this model, the temporal decline of immunity would lead to more pandemic ‘waves’, but for a fixed disease transmissibility this would not alter the maximal height of the peak number of infected, since this occurred for all the initially susceptible population. Finally, we note that this work does not include a vast range of divisions for the population, including age, socio-economic status etc. We have not included this directly, since we wanted to estimate as cleanly as possible how the heterogeneity in the contact pattern, arising from a geographically distributed population, could affect the evolution of a disease. We are aware that for instance the distribution of age has an enormous impact on the health risk and that this risk is vital in the prediction of hospitalizations in modern society. However, our aim was to understand the bias in the prediction of a disease, based on the data that comes during the early periods of a disease, independently of the mortality of this disease. Mathematical predictions of disease progression have been heavily criticized [38,39] and it is important to improve the theoretical foundations of the mathematical descriptions, in order to increase the confidence in the predictions. Our work highlights the importance of estimating the spatial clustering and connectivity skewness in the population in order to correct the predictions based on SEIR models, by quantifying their biases from not including spatial clustering. We hope that this work could serve as an input to the modelling and prediction of future pandemics and the importance of avoiding super-spreaders in high-density areas.

### 3.1. Methods

#### 3.1.1. Construction of spatial network

We initialized *N*_0_ agents on a network generating a total of *μ* × *N*_0_ links between two agents, with an assigned interaction strength *β*_*ij*_ for each link. The average contact number, *μ*, was fixed to 20, based on results from the Danish HOPE project, gathering data on population behaviour since April 2020 [34]. In order to include a realistic, geographical distribution of the population, we randomly selected agent locations from a two-dimensional kernel density estimate we had generated based on housing sales in Denmark 2007–2019 (data given with permission from Boligsiden, [33]). We note that in this distribution, we do not take specific geographical elements such as roads or environment into account (which has been previously studied for other diseases [40]) as we assume that this effect is small in a country like Denmark, where all parts are connected and natural obstacles such as mountains and rivers are not present. To connect the agents, we used a hit and miss method, where two random agents are first suggested and then connected with probability, $p(d)=e^{-\rho \cdot d_{ij}}$. Here, *d*_*ij*_ is the distance between agents and *ρ* is a parameter with units of inverse distance. We choose *ρ* = 0.1 km^−1^ (i.e. 10 km) which is the average distance travelled by labour force (statistics Denmark [23]). To allow some long-distance interactions, we introduced a parameter $\epsilon_{\rho }=4\text{\%}$ representing the fraction of distance-independent connections. This value we based on the fraction of workers travelling longer than 50 km to work (statistics Denmark [23]).

#### 3.1.2. Fits and predictions

We defined an early phase to be the period of time when between 0.1% and 1% of the population were infected (blue lines figure 3*a*). We then fitted *β* and a time delay, *τ*, to the SEIR model with a *χ*^2^-fit (assuming Poissonian statistics) and kept *λ*_*E*_ and *λ*_*I*_ fixed to the true numbers (used in the simulation). The initial number of infected, *N*_init_, was also fixed to the true numbers. The fit parameters were then inserted into the SEIR model, and $I_{\mathrm{peak}}^{\mathrm{fit}}$ and $R_{\infty }^{\mathrm{fit}}$ were extracted from the fitted model and compared to the $I_{\mathrm{peak}}^{\mathrm{ABM}}$ and $R_{\infty }^{\mathrm{ABM}}$ from the ABM simulation.

## Data Availability

Data and relevant code for this research work are stored in GitHub: www.github.com/ChristianMichelsen/NetworkSIR and have been archived within the Zenodo repository: https://zenodo.org/badge/latestdoi/258223118.
